# Merkel Cell Polyomavirus and Merkel Cell Carcinoma, France

**DOI:** 10.3201/eid1409.080651

**Published:** 2008-09

**Authors:** Vincent Foulongne, Nicolas Kluger, Olivier Dereure, Natalie Brieu, Bernard Guillot, Michel Segondy

**Affiliations:** University of Montpellier I, Montpellier, France

**Keywords:** Merkel cell carcinoma, Polyomavirus, MCPyV, letter

**To the Editor:** Merkel cell carcinoma (MCC) is a primary cutaneous neuroendocrine tumor. This aggressive skin cancer is uncommon but increasing in frequency. During 1986–2001, incidence rate tripled; average annual increase was 8% (*1*). MCC shares epidemiologic features with Kaposi sarcoma, a malignant tumor associated with human herpesvirus 8 infection (*2*). In particular, MCC affects predominantly immunocompromised patients such as organ transplant recipients (*3*,*4*), patients with B-cell lymphoid tumors (*5*), and patients with AIDS (*6*). This similarity between MCC and Kaposi sarcoma may support the hypothesis of an infectious origin of MCC.

A new polyomavirus, provisionally named Merkel cell polyomavirus (MCPyV), has been recently identified in tumor tissue from patients with MCC. Furthermore, clonal integration of viral DNA within the tumor genome was observed in most of the cases (*7*). To assess the implication of MCPyV in MCC, we tested tumor biopsy samples collected from 9 patients with MCC. Patient median age was 65 years, and 2 patients were immunocompromised (patient 1 had a lymphoma, which was treated with rituximab; patient 7 had psoriatic rheumatism, which was treated with corticosteroids and methotrexate). As controls, biopsy samples from 15 patients with diverse proliferative or inflammatory skin or mucosa lesions were tested (Table).

**Table:** Merkel cell polyomavirus from 9 patients with Merkel cell carcinoma and 15 control patients, France*

Patient no.	Age, y	Sex	Diagnosis	Sample	PCR primer pair		
					LT1	LT3	VP1
1	57	M	MCC, primary	Fresh biopsy, buttock	+	+	+
2	61	F	MCC, primary	FFPE biopsy, buttock	–	+	–
3	57	F	MCC, primary	FFPE biopsy, lower eyelid	–	+	+
4	82	F	MCC, primary	FFPE biopsy, cheek	–	+	–
5	78	M	MCC, metastatic	Fresh biopsy, jugular lymph node	–	+	+
6	80	F	MCC, primary	Fresh biopsy, upper eyelid	–	+	+
7	65	M	MCC, primary	FFPE biopsy, temple	–	+	–
8	81	M	MCC, primary	FFPE biopsy, forearm	–	–	–
9	60	M	MCC, primary	FFPE biopsy, forearm	–	+	+
10	61	M	Hyperkeratosis	Fresh biopsy, foot	–	–	–
11	49	M	Seborrheic keratosis, penis	Fresh biopsy, penis shaft	–	–	–
12	60	M	Nonspecific lesion, esophagus	Fresh biopsy, lesion	–	–	–
13	40	M	Nasal papilloma	Fresh biopsy, papilloma	–	–	–
14	58	M	Anal condylomas	Fresh biopsy, condyloma	–	–	–
15	44	M	Epidermodysplasia verruciformis	Fresh biopsy, skin	–	–	–
16	19	M	Cutaneous warts	Fresh biopsy, wart	–	–	–
17	57	F	Cutaneous warts	Fresh biopsy, wart	–	–	–
18	64	F	Cutaneous nodule	Fresh biopsy, skin	–	–	–
19	6	F	Pharyngeal papillomatosis	Fresh biopsy, pharynx	–	–	–
20	27	M	Vocal cord polyp	Fresh biopsy, polyp	–	–	–
21	58	M	Lichen	Fresh biopsy, skin	–	–	–
22	63	M	Skin inflammation	Fresh biopsy, skin	–	–	–
23	72	M	Skin inflammation	Fresh biopsy, skin	–	–	–
24	57	M	Skin inflammation	Fresh biopsy, skin	–	–	–

*MCC, Merkel cell carcinoma; +, positive Merkel cell polyomavirus PCR amplification; FFPE, formaldehyde-fixed paraffin-embedded; –, negative Merkel cell polyomavirus PCR amplification.

DNA was extracted from fresh tissue samples by using the QIAamp DNA Mini Kit (QIAGEN, Courtaboeuf, France) according to the manufacturer’s instructions. Paraffin was removed from previously formaldehyde-fixed, paraffin-embedded biopsy samples with xylene, and the samples were rehydrated with decreasing concentrations of ethanol. The extracts were tested for MCPyV DNA by PCR using 3 sets of primers initially described by Feng et al. (*7*) to target the predicted T-antigen (LT1 and LT3 primer pairs) and the viral capsid (VP1 primer pair) coding regions. Extracted DNA (5 μL) was added to 45 μL of the reaction mixture, which contained 5 μL 10× PCR buffer, 10 μL 5× Q-solution (QIAGEN), 2.5 mmol/L MgCl_2_, 200 μmol/L each dNTP, 2.5 units Taq DNA polymerase (QIAGEN), and 15 pmol of each primer. Touchdown PCR conditions were as follows: 95°C for 5 min followed by 35 cycles of denaturation at 95°C for 30 s; annealing at 61°C (10 cycles), 59°C (10 cycles), and 57°C (15 cycles) for 30 s; extension at 72°C for 1 min; and a final extension step at 72°C for 10 min. Amplification products were subjected to electrophoresis in a 2% agarose, 1× Tris-borate-EDTA gel stained with ethidium bromide and examined under UV light. The sizes of the fragments amplified with the LT1, LT3, and VP1 primers pairs were 439, 308, and 351 bp, respectively. A negative control was included in each experiment; positive samples were confirmed by analyzing a second stored sample aliquot, and the amplified fragments were sequenced by using the same primers used for the amplification. The sequences were submitted to GenBank under accession numbers AM992895–AM992906. Total DNA level in sample extracts was measured by using the LightCycler control DNA kit targeting the β-globin gene (Roche Diagnostics, Meylan, France).

MCPyV sequences were detected in 8 of the 9 patient samples and in none of the control samples (Table). Results for all 8 patients were positive with the LT3 primer pair, whereas they were positive for only 5 with the VP1 primer pair and only 1 with the LT1 primer pair (Table). Because the LT1, VP1, and LT3 primer pairs generate the longer, intermediate, and shorter DNA fragments, respectively, the difference in sensitivity could result from the deleterious effect of formaldehyde fixation on DNA; this effect would increase with the length of the fragment to be amplified. The negative result obtained for patient 8 might suggest that some MCC patients are not infected with MCPyV. This explanation is in accordance with the findings of Feng et al., who reported 80% prevalence of MCPyV in patients with MCC (*7*). Nevertheless, the single negative result observed in our study might alternatively be explained by insufficient tissue or by DNA degradation through the fixation and embedding process. Indeed, the level of β-globin gene DNA was much lower in the sample from this patient (441 pg/µL) than in samples from the other patients (median 13,500 pg/µL, interquartile range 8,902–19,750 pg/µL).

As observed with human papillomaviruses, a gene disruption caused by viral DNA integration into the host genome might be an alternative hypothesis to explain the lack of amplification of an MCPyV genome region (*8*). Sequences of the amplified PCR product were 99%–100% identical to those reported by Feng et al. (*7*), which indicates that this virus is genetically stable.

In summary, we detected MCPyV DNA sequences in 8 of 9 tumor samples from patients with MCC but in none of 15 control samples. Our results confirm the likely association of MCPyV with MCC. The epidemiologic characteristics as well as the carcinogenic role played by this newly discovered virus need to be more thoroughly investigated.
